# Understanding patient preferences for emergency care for lower triage acuity presentations during GP hours: a qualitative study in Australia

**DOI:** 10.1186/s12913-022-08857-8

**Published:** 2022-11-29

**Authors:** Viola Korczak, Kenneth Yakubu, Blake Angell, Paul Middleton, Michael Dinh, Thomas Lung, Stephen Jan

**Affiliations:** 1 The George Institute for Global Health, University of New South, Sydney, Australia; 2 The Green Light Institute for Emergency Care, Royal Prince Alfred Hospital, Camperdown, Australia; 3 Faculty of Medicine and Health, The University of New South, Sydney, Australia; 4 South Western Emergency Research Institute, Ingham Institute, Liverpool, Australia; 5 South Western Sydney Clinical School, University of New South Wales, Sydney, Australia; 6 NSW Agency of Trauma and Injury Management, PRISM, Agency for Clinical Innovation, St Leonards, Australia

**Keywords:** Emergency department, Health policy, Models of care

## Abstract

**Background:**

Low acuity presentations to Australian emergency departments drive long wait times, higher costs and may be better treated in primary care settings. This study sought to understand factors leading these patients to present to emergency departments.

**Methods:**

Semi-structured interviews were carried out with patients at two tertiary emergency departments in Sydney during general practitioner opening hours. Nvivo was used to code the interviews and a thematic analysis was carried out to capture the main themes from the interviews.

**Results:**

Forty-four interviews were included in the analysis across the two sites. They represented a diverse population in terms of ethnicity, education and socioeconomic background. Patient preferences for emergency care were organised into four main themes: (i) patients were referred (either by another health service, work, friend, or family), (ii) emergency department factors (convenience of investigations and severity of symptoms requiring emergency care), (iii) GP factors (does not have a GP, cannot find an appointment with a GP or has previously had a negative experience with a GP) and (iv) personal factors such as their connection to the hospital.

**Conclusion:**

Multiple factors led patients to seek ED care for low acuity presentations during GP hours. Some of these factors could be addressed to meet patient needs in the community, however this is currently not the case. Addressing these factors to improve access to GP clinics and the availability of services outside the hospital setting could reduce ED presentations and likely improve patient experience.

## Background

There is increasing strain on public hospital emergency departments. Between 2014 and 2015 there was an annual growth of presentations to emergency department (ED) of 3.2%, however this increased to 6.9% in 2020–2021 in Australia [1]. This trend has also been evidenced in other parts of the world [2], causing increased pressure on health systems. ED crowding is a common theme across different health systems, even in countries with universal healthcare, though less so in Scandinavian countries [3]. It is important to understand the reasons for presentation so that models of care can be developed which best meet the needs of patientsA study by Toloo et al. examined GP type presentations to a metropolitan hospital in Brisbane over 3 years found that the number of GP type presentations to the ED varied depending on the method used to measure these presentations [4]. This varied from 27% using the Australian Health and Welfare Institute metric, verses 6% using the Australasian College of Emergency Medicine (ACEM) measure to 8% using the Spivulis method [4]. Dinh et al. undertook a retrospective analysis of 11 million presentations over an 11 year period in the greater Sydney area and found that 40% of presentations could be classified as GP presentations and that GP presentations to ED increased across all age groups during the study period [5]. Regardless of the number of GP presentations to the ED, there is a cohort which could be reviewed in the community rather than the ED.

Discrete choice experiments have been undertaken in the UK [6] and Singapore [7] to explore the factors influencing patient decisions to present to the ED. A systematic literature review also examined patient preferences for emergency care but most of the papers were from the US and UK [8–10]. An Australian study examined patient preferences for emergency care but looked specifically at an older cohort of patients over 70 years of age [11]. An Australian Institute of Health and Welfare (AIHW) report on the use of emergency departments found that the majority of people who visited the ED rather than the GP did so because they were taken to the ED by an ambulance, their condition was serious or they were referred by their GP [12]. Wong and Hall found that patients with a better GP experience are less likely to visit the ED, suggesting that improving GP quality could reduce ED use [13]. Similar issues were raised in the rural setting [14]. Outside of these studies, however, the complex factors influencing patient decisions to present to ED rather than primary care have not been well explored. Morley et al. showed that chronic conditions are a driver for ED presentations but that local factors should also be examined [15].

This study sought to understand patient preferences and care seeking behaviour for ED care for lower acuity presentations during GP opening hours by undertaking interviews with patients at two tertiary emergency departments. The aim of the study was to understand patient preferences for care so that models of care can be developed which best meet the needs of patients.

## Methods

### Study site

This was a qualitative study using semi-structured interviews with patients at two tertiary emergency departments in Sydney. Hospitals A and B are both level six tertiary hospitals, major trauma hospitals with ICU and cardiothoracic departments situated in metropolitan areas. Hospital A is in the inner city and Hospital B is in southwestern Sydney. Both sites were chosen as they have different demographic profiles [16, 17] to ensure a range of views were represented.

Ethics was approved by Sydney local health district (X19–0228 and 2019/ETH10574). Specific applications (SSA) were approved at both sites before the study was carried out.

### Study design

The questions in the interviews were informed by reviewing the literature on the topic and VK’s clinical practice and experience as a clinician working in the ED. The interviews began with demographic questions, then moved to patient factors and their time in Australia (if they were from overseas). Patients were asked if they used primary care in the community and their relationship with a GP. Patients were then asked why they chose the ED over the GP that day. They were also asked questions around accessing the ED and their experience of the ED and whether they considered any other options before presenting to the hospital.

The semi- structured interviews were carried out by VK (qualifications in economics, public health and medicine). The interviewer introduced themselves to the participants, explained the purpose of the study, answered any questions the participants had and obtained written consent. Interviews were continued until data saturation was achieved [18, 19]. The COREQ checklist was used to report findings [20].

### Inclusion and exclusion criteria

All adult patients with a triage category of 3, 4 or 5 were included who were in the waiting room between 9 am and 5 pm on weekdays. Triage 1 is life threatening and needs to be seen immediately, triage 2 should be seen by a doctor within 10 minutes, triage 3 within 30 minutes, triage 4 within 60 minutes and triage 5 within 120 minutes [21].

If patients did not speak English telephone interpreting services were available. If a telephone interpreter was used, this interview was recorded and then later the English translations transcribed as per the other interviews to ensure the inclusion of culturally and linguistically diverse populations. Patients were excluded if they were: paediatric (less than 18 years old), brought in by ambulance or referred by a GP. They were also excluded if they had suicidal ideation or presented with vaginal bleeding, as these were considered potentially sensitive presentations and it was thought to be inappropriate to approach patients.

### Sampling

Participants were selected by convenience sampling with all patients who met the inclusion criteria during the time the researcher was conducting interviews, invited to participate. Interviews were carried out at the two sites in February and March 2022. All the interviews were carried out face to face.

### Analysis

These interviews were recorded and then transcribed verbatim by a professional transcribing service. The interviews were checked by VK who listened to all the audio and crosschecked the text transcription. VK also kept field notes during the interview process and reflected on their experience as a clinician undertaking a qualitative study and how that may impact on interactions with patients, in a dynamic different to the one they were used to (as a researcher rather than a doctor in a patient interaction).

The study used an inductive approach [22] and undertook thematic analysis. Six phases of thematic analysis were undertaken [23] including familiarising oneself with the data, generating initial codes, searching for themes, reviewing themes, defining and naming themes and producing the report [23]. Two coders (VK and KY) coded the data in Nvivo 12 Pro and another co-author (TL) was available for discussion if there were any differences that could not be resolved. Initially a subset of interviews was coded and the coders met to discuss their respective codes. They proceeded to code an additional 10 interviews and met again to discuss the coding framework. Once initial themes were captured and the coders had agreed, VK coded the remaining interviews using the developed framework. This was an iterative process and VK read and coded the transcripts multiple times, whilst reflecting on the individual interviews and notes from their field journal. ‘Peer-check in’ was carried out with an external academic who was not a part of this project but has extensive experience in thematic analysis. ‘Member check-in’ was not possible as the patients were from the waiting room in the two respective emergency departments and could not be contacted again.

## Results

### Demographics

In total, 49 interviews were conducted at two hospitals (29 interviews at Hospital A and 20 interviews at hospital B). During the course of the interviews 3 patients at hospital A and 2 patients at hospital B revealed that they were in fact referred by their GPs and were excluded from the final analysis. This left 44 interviews which were included in the analysis, 26 interviews from Hospital A and 18 from Hospital B. Table 1 summarises the main demographics of the participants at the two sites. To ensure anonymity of the participants, the demographics were combined into one cohort.

**Table 1:**
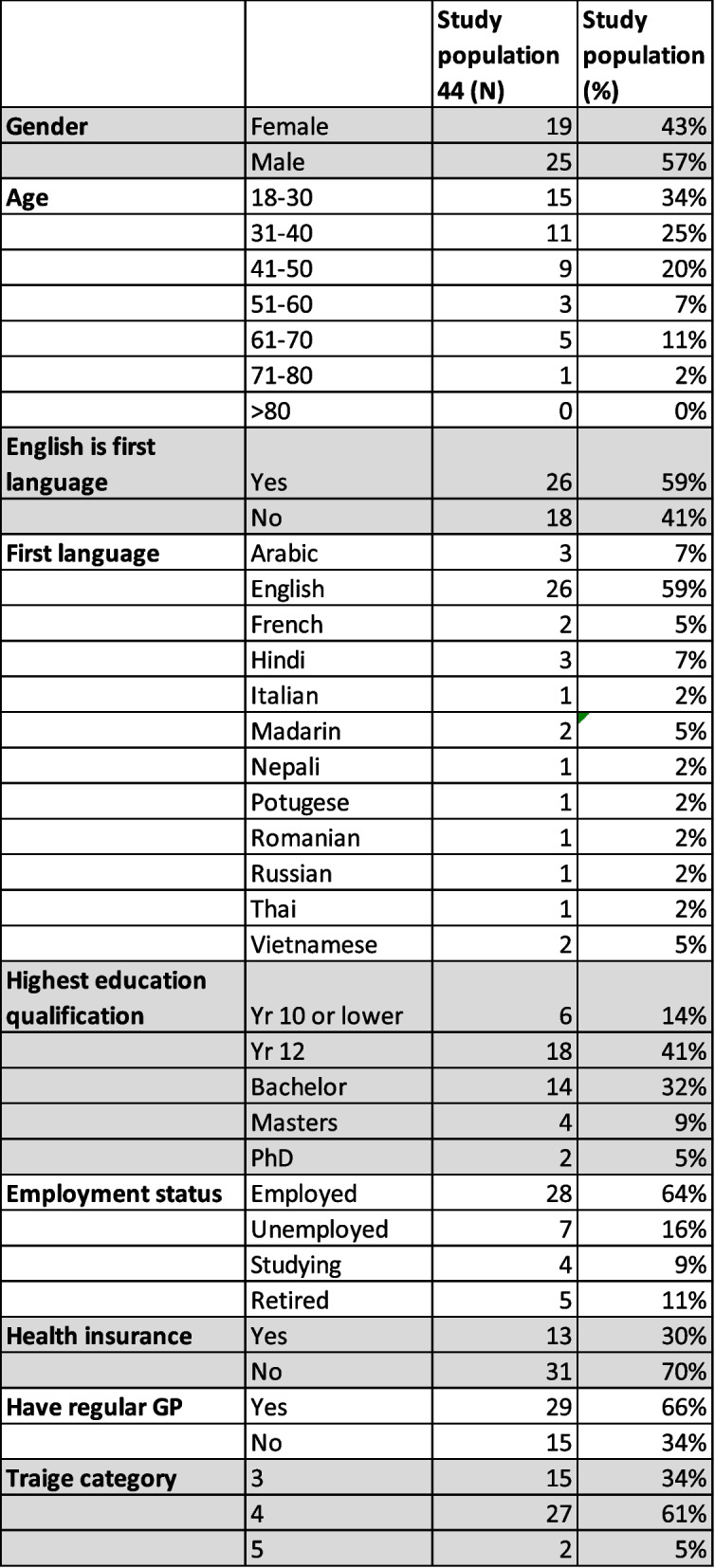
Patient demographics.

Most of the presentations were triage category 4. Fifty sever percent of the cohort was male, and 34% were between the ages of 18 to 30 years old. One interview was conducted with an interpreter, and 41% of participants spoke English as a second language. In total 11 primary languages other than English were represented in this cohort, with Arabic, Hindi, French and Mandarin the main languages spoken other than English. The sample of patients included a diverse group, not only in terms of language but also in educational qualifications and included those who had year 10 qualifications or lower (14%) to those with a PhD (5%). The majority of study participants were employed (64%) but did not have private health insurance. Sixty six percent stated they had a regular GP.

### Thematic analysis

We identified four main themes driving patient preferences for the emergency department care for lower triage acuity care during GP hours. Table 2 summarises the themes and subthemes.

**Table 2:**
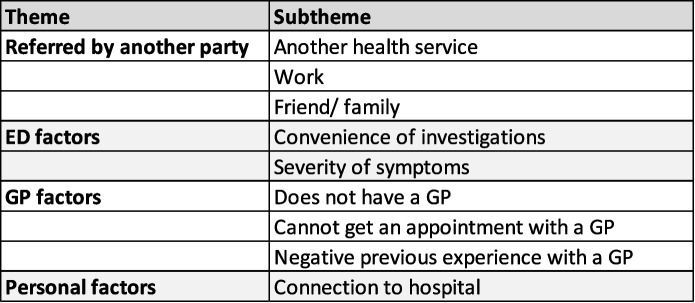
Main themes driving patient preferences for ED care.

### Referred by another party

#### Another health service

None of the patients in the study were brought in by ambulance or referred by a GP who had reviewed them. Some patients stated they were referred by the GP after not being able to secure an appointment, but these patients were included as they had not specifically been referred after a clinical review. Some patients presented to the ED because they were referred by a third party. HealthDirect [24] is a free 24 hour service which can be accessed online through a ‘symptom checker’ or patients can call a 1800 number to speak to a nurse for advice. There were multiple examples of patients accessing this service and then presenting to the ED after being given advice to go to the hospital:

“…….. called the nursing helpline and they told me to come in straight away” (Hospital A, #88, triage 4)

A patient described the service and process of calling HealthDirect:

“yeah, HealthDirect….. and that was just to see if I was warranted to actually go into emergency. Um, yeah, because I didn’t want to like, waste anyone’s time. I was like if the GP can - can sort it out, then I’ll just go to them”. (Hospital A, #105, triage 4)

#### Work

Similar to HealthDirect is a service for workplace injuries. Injurynet [25] is a private provider and one patient explained his experience in using the service and what brought him to the ED:

“yeah so there’s like a triage nurse on the other end, ah, you give them your details, tell them what happened, like take a photo or two, send it to them and they go, oh, yeah, you’re going to - you may need stitches, maybe not. You can go to a clinic; you can go to a hospital. The hospital’s closer. That’s how they do it”. (Hospital B, #116, triage 4)

For smaller workplaces that may not have access to Injurynet, or other private providers like it, others were referred directly by their workplaces:

“and then I went- came in this morning and my boss went “hang on a minute. I had this happen to me, um, it’s really more serious than you think. Um, you don’t want to get an infection”. So he sent me here”. (Hospital A, #95, triage 4)

Some patients expressed a reluctance to present to the ED after a workplace injury but because it was witnessed by a supervisor, were encouraged to go to the ED:

“ah, I was working near a, like my supervisor was there and goes, “oh you should probably get that looked at”. I went, yeah, all right, get it looked at”. (Hospital A, #97, triage 4)

Occasionally a patient called the emergency department of the hospital for advice as to whether they should present to the ED. In these situations, patients are never turned away but rather told to present to the ED ‘if they are worried’. Patients will often interpret this as being asked to present:

“……. I dialled the number, um, to the hospital and they say like come to the emergency room instead. So I didn’t consider any option….” (Hospital A, #79, triage 5)

#### Friend or family

When asked why they presented to the ED, other patients stated they were encouraged to do so by family or friends:

“oh, my girlfriend” (Hospital A, #99, triage 4)

Another patient was referred by her friend who is a nurse:

“but then my friend who’s a nurse looked at it and said ‘go to the emergency, that’s really dangerous’”. (Hospital A, #88, triage 4)

### ED factors

#### Convenience of investigations

There are also ED factors driving patient preferences for emergency care. The main themes are around convenience of the ED and severity of symptoms. The convenience of having all investigations in one place and performed straight away, especially imaging and blood tests, was an incentive to present to the ED. This was a recurring theme in the interviews:

“……no. Just, ah, the common sense. Because if I go-go to my GP, he’s going to refer me to the hospital or refer me to a specialist. But if I go here they do all the scan and that is, like- it’s quicker. All the tests and scan and they decided what’s the problem. If something need operation if something need medications. Yeah”. (Hospital B, #118, triage 3)

The convenience of investigations was a strong and recurring theme across both hospitals:

“So when I come here it’s a straight-out answer”. (Hospital B, #125, triage 4)

One patient described the time saving nature of presenting to the ED rather succinctly:

“Uh, I just, kind of, thought, fuck it, I may as well here- go here instead of having to go to the doctors. Call them up, all that stuff. It’s just easier”. (Hospital A, #103, triage 4)

This convenience also extended to appointments with specialists but this was only raised at Hospital B, which serves a lower socio-economic population:

“….I think it may be quicker than I waiting for specialist. I don’t know”. (Hospital B, #119, triage 3)

#### Severity of symptoms

Another factor driving patient preferences for emergency care is patients’ symptoms and patients believing their symptoms needed hospital rather than community-based care:

“……. the potential- potential impact…… that things-like not-I’m not a doctor but things like sepsis, blood poisoning, that kind of thing, would always be a worry”. (Hospital B, #114, triage 3)

Patients were making decisions based on their past experiences with the healthcare system:

“because this one is recurrence, then I generally just go straight to the hospital for this case. Um, any other time I would go to the GP”. (Hospital B, #129, triage 4)

Based on previous presentations which needed hospital treatment, they were rightly concerned that the same symptoms may again need hospital intervention:

“Because, um, the pain I got on the back of my leg, and I had this before and I had to be operated”. (Hospital A, #78, triage 4)

Patients also expressed concern that their symptoms were progressing and needing hospital treatment:

“well, the bite was on Sunday….. initially for the first couple of days it was very localised around the bite….and then yesterday it just rapidly spread….. and it was the rate of progression of the spread of infection…..and I just took the decision that I needed to get to the ED immediately”. (Hospital B, #114, triage 3)

### GP factors

There are three main GP factors which contribute to ED presentations. The main themes captured were those patients who either did not have a GP, could not get an appointment with their GP or had a negative experience previously with their GP.

#### Patients who do not have a GP

The reasons for not having a GP are multiple. Several patients had the same GP since childhood but due to moving away for study or work no longer had a GP and found it difficult to find a new one:

“……. I’ve just moved into the inner-west so, um, I’ve lost my regular GP of 36 years”……….No, I’ve actually struggled to find one. No-one wants to accept new patients ..….you know, friends and people that live here, they recommend people. As soon as you call, they basically don’t want to take new patients. So you’re left with bulk billing, um random, walk in places”. (Hospital A, #82, triage 3)

Other patients are travelling and therefore do not have a regular GP, such as this patient who came to Australia to visit her family:

“I don’t have a doctor here because I only came as a tourist and only staying for two months”. (Hospital A, #87, triage 3)

While others have moved to Australia but do not understand the health system as it is different to where they grew up:

“So, first of all, the concept is new. Like, I heard, like somebody talk, oh this is my GP… and I don’t know how to have a regular GP. Like is it a process or you just keep, randomly, like regularly going to that person”. (Hospital A, #91, triage 4*)*

Or they were guided by family members who navigated the Australian health care system on their behalf. In this case, the patient’s wife told him where to present:

“So, no, I- I really don’t understand the private thing, the medicare thing. I just take my orders”. (Hospital A, #108, triage 4)

While others are young and healthy and do not feel they need a regular GP and the continuity of care that offers:

“Haven’t really looked yet. I’ve never had a GP. Like a dude that I just see, I think”. (Hospital A, #102, triage 4)

#### Unable to book an appointment with a GP

Even those patients who do have a GP, it is often difficult to book an appointment, which is why they present to the ED. There were multiple examples of this in the interviews:

“And I got up this morning. Went to three different medical centres in Newtown. No-one would see me”. (Hospital A, #101, triage 4)

Others could not access their GP on particular days of the week:

“and I know today- well, today is Thursday and my doctor is not in today”. (Hospital B, #117, triage 4)

Waiting time at the GP was also a factor:

“Okay. The reason why I came to the emergency because when I had an appointment with my daughter’s GP, I had to make an appointment. The first one available was in a month’s time. So in the meantime, the pain was unbearable so I had to get something done”. (Hospital A, #87, triage 3)

#### Negative experience with a GP

Others presented to the ED because they had a negative experience with a GP previously. This was usually around GP time constraints. Short consultation times with GP was a recurring theme which contributed to an overall negative experience:

“Ah, I wasn’t very happy because she always was very, very busy. She never had time to see the patients”. (Hospital A, #78, triage 4)

The responses showed that GPs are also under pressure to see many patients:

“and it’s, uh, it’s more better than GP. Because of-every time we go to GP, we have [interruption] waiting for, yeah, about half an hour. Sometimes one-hour, long time. And they see the GP. And the GP just, uh, spend, uh, five minutes. Finish. Yeah. So not- it’s, uh, very bad experience sometimes”. (Hospital A, #100, triage 3)

Others were seeking a second opinion:

“It really seems, sort of, I don’t know, I think he got it wrong”. (Hospital B, #119, triage 3)

And this happened across both hospitals:

“….. because she did say that it would be even better for me to come here, to have a better test. And, uh, to see if any specialist could have [interruption] tests say something, because she doesn’t really have a clue of what’s happening”. (Hospital A, #93, triage 4)

### Personal factors

There were also personal factors which were drawing patients towards the ED. They had a connection to the hospital. For some it was because they already had an established relationship with that particular hospital:

“And the message said to come here. And this is where- they’ve saved my life here so many times. They’ve got my record. I-I come – I came straight away”. (Hospital A, #104, triage *3)*

Many patients also appreciated the care they were given in the ED by paramedics, nurses and doctors. This quote is from a patient who reported seven presentations to the ED in the previous year:

“I feel like- I feel sorry for the nurses because it’s very busy. I don’t know how they keep up, and that lovely lady brought my Panadol, but…other than that, I can’t fault it. The man out the front in the red shirt that does the triage part, every time he’s been lovely, he’s a lovely man” (Hospital B, #125, triage 4)

The ‘man in the red shirt’ is the patient experience officer in the waiting room and many people commented on and appreciated that role in the waiting room at both hospitals:

“…someone at every point pretty much. At the door, telling you to go to the – the office, where to sit. And they, uh, diagnose you from there, sit somewhere else. The person coming in telling you where this is what we have in the thing. If you have a bathroom, here, chargers, everything like that….” (Hospital A, #98, triage 4)

For others, it was that they did not feel judged when they sought care there, also because they had a history at that hospital:

“yeah, a bit like that. They also know that I have addiction problems and they don’t really rub that in, kind of thing, or make me feel, um, I don’t know, like, I’m wrong or I’ve got something wrong”. (Hospital B, #125, triage 4)

This connection was due to a few factors including a sense of community ownership, which was evident at both hospitals:

“Oh, I only come to this hospital because it’s the only hospital I trust”. (Hospital B, #117, triage 4)

This was a sentiment that was repeated by multiple people:

“…nice, very polite. Really I think [hospital B] is the best…I think it’s the best hospital for the people”. (Hospital B, #121, triage 4)

It was a theme which was evident at both hospitals:

“…I totally relax as soon as I walk in here…I know it’s old. It’s not like a great private hospital. I know that. I- I have private cover. Here is where I will come….”. (Hospital A, #104, triage 3)

The emergency department provides a safety net, but there is also a sense of community ownership and pride in the respective hospitals which makes patients feel comfortable.

## Discussion

Four main themes were identified concerning why patients present to the ED during GP opening hours for low triage acuity presentations. Firstly, they are often referred by HealthDirect, work, friends or family. The second theme was ED factors, which included having the convenience of investigations in one location with immediate results. Another sub theme within ED factors was severity of their condition which they believed needed hospital rather than community care. The third theme identified from the interviews included GP factors such as not having a GP, not being able to secure an appointment or having a negative experience with a GP previously. The fourth theme identified personal factors linking patients to the respective hospitals.

The reasons for presenting to the ED for low triage acuity presentations during GP hours are multifactorial. ED factors, in particular the convenience of investigations, was a driving factor. Patients generally liked having a diagnosis at the point of care. They were also more likely to present to the ED if they thought that their symptoms needed hospital rather than community care, despite the lower triage. These concerns were often justified as a lower acuity triage may still need hospital treatment, for example, intravenous antibiotics for a spreading cellulitis infection.

Some patients presented because they were seeking reassurance, or sometimes a second opinion. NSW Health has attempted to address this through the ‘keep emergency departments for emergencies’ [26] campaign. These are posters adopting a traffic light approach and encouraging patients to call HealthDirect in the first instance, go to a pharmacy for minor ailments, GP for more chronic issues and the ED for life threatening emergencies. There is scope to redirect patients to more appropriate care and it is clear from the interviews that HealthDirect is widely used. None of the patients interviewed mentioned that they considered pharmacy as an option prior to coming to the ED. However, several patients mentioned calling HealthDirect before coming to the ED.

Multiple factors drive patients towards the ED. A lack of understanding of the Australian health care system was identified. The patients interviewed represented a culturally and linguistically diverse cohort. This also reflects Australia’s multicultural population with 27.6% of the population being born overseas and 48.2% having a parent who is born overseas [27]. Several of the patients who had a GP, and who spoke English as a second language, had a GP who spoke their primary language and the medical consultation with the GP was carried out in this primary language. Any policies which seek to link patients into primary care should consider the multicultural nature of Australia’s society and develop policies which are culturally inclusive and appropriate.

For those patients who are not confident navigating the Australian healthcare system, there is scope to provide public health messaging to improve health literacy. For example, this could include more messaging about the importance of primary care and of having a GP, and explaining the Australian healthcare system and tailoring the messages to people who speak English as a second language. The messaging about the Australian health care system could be tailored for international students and in immigration as people are entering the country.

Likewise, the pathway to linking patients to GPs could be more specific. HealthDirect offers several services including an online ‘symptom checker’, a telephone hotline with a triage nurse, and also a ‘GP finder’ page on the website. The ‘GP finder’ includes a postcode locator and some other variables such as whether a practice is bulk billing or has wheelchair access. Other variables could be included such as whether the GP is currently accepting new patients, or if the GP speaks other languages and can offer a consultation in another language, or whether they have a special interest in mental health or paediatrics. This would help patients find a GP which would ‘match’ their needs, and more easily link into community care.

However, to offer more GP services, medical workforce shortages need to be addressed. The supply of a medical workforce has been on the agenda for some time in Australia. There is a chronic shortage of general practitioners and this is not a new problem [28]. Australia continues to rely on overseas trained medical doctors to fill deficits in the Australian health system [29]. Apart from posing ethical issues around recruiting Australia’s medical workforce from neighbouring countries it highlights that workforce planning needs greater policy attention. In particular, GP shortages are particularly problematic for those living in rural areas [30] but even in the inner-city area this was identified as an issue in the interviews.

These pressures on general practitioners results in time constraints and shorter consultations, which appears to drive patients to the ED. As one patient explained, she can wait an hour for the GP for a 5-minute consultation but prefers to wait 2 or 3 hours in the ED, be reviewed by a doctor for a longer time and have all her investigations at the point of care. GP workforce pressures appear to have a flow on effect in the ED, especially shorter consultations with the GP. This supports work by Wong and Hall that a negative experience with the GP can increase ED care seeking [13].

Waiting time in the ED did not seem to be a deterrent for these patients seeking ED care. This is likely because patients expect to wait and had made the conscious decision to attend regardless of the expected wait time. Out of pocket costs did not seem to influence preference for the ED or care seeking behaviour either. All the patients in hospital B had bulk billing GPs and did not have out of pocket costs. The lower socio-economic area of hospital B may likely be driving billing practices.

Investing in the health workforce is one solution but has a long lead time. Other shorter-term measures could include focusing on policy and remunerating General Practitioners in line with their hospital colleagues. Another approach would be to hold a national ‘health summit’ to further explore how Medicare could be strengthened. In the shorter term, the Government has also committed to increasing the number of urgent care centres [31], however it remains to be seen how these will be staffed. As articulated by Wilson et al., “a whole of system approach” is needed to realign policy priorities in this setting [32].

It was interesting that patients felt such community ownership of both hospitals. They had overwhelming trust in the health system, and this was evident in the quotes which came from the interviews. Although it was not a direct question in the interviews, it was clear that the patients felt both pride and trust in both hospitals. This could be leveraged to provide more efficient services in the ED or branding new clinics to see low acuity patients as part of the hospital.

### Strengths and limitations

A major strength of the research was the diversity of the group of patients that were interviewed in terms of gender, cultural background, language and educational qualifications. The patients were from two local health districts and represented a diverse group which added to the richness of the analysis. Another strength was the number of interviews and the robustness of the coding. Having two coders, from different professional backgrounds, offered a richer interpretation of the data.

A weakness of the study was that the interviews were relatively short in duration, with the meantime of the 44 interviews being 7 minutes. This was likely due to the straightforward nature of questions. We were able to achieve thematic saturation, increasing confidence that we managed to elicit the key factors driving the decisions of these patients. There were five patients who, during the course of the interview, stated that they were advised to attend the ED becuase they were referred by the GP, however this was not in the triage note. The interviewer made every effort to reassure patients that they did the right thing in presenting to the ED but the question itself may have made some people feel uncomfortable and some may have said that they were referred by the GP when they were not. Any patient who stated that they were referred by the GP was subsequently removed from the analysis. Also, although interpreting services were available, only one patient was interviewed with an interpreter. Despite this, the sample includes a culturally diverse cohort.

## Conclusion

Four main themes were found to drive patient preferences for ED care for low acuity presentations during GP hours: being referred by a third party, ED factors, GP factors or personal factors and their connection to the hospital. There is scope to improve the current system to meet the needs of patients outside of the ED by improving access to GPs. This primarily needs to be done by addressing workforce shortages and pressures on General Practice. It could also include improving services to connect patients to find their GP ‘match’ and use more inclusive health promotion messaging which reflects Australia’s multicultural society.

## Data Availability

The datasets generated and/or analysed during the current study are available from the corresponding author on reasonable request.
